# Regulation of Aging and Longevity by Ion Channels and Transporters

**DOI:** 10.3390/cells11071180

**Published:** 2022-03-31

**Authors:** Kartik Venkatachalam

**Affiliations:** 1 Department of Integrative Biology and Pharmacology, McGovern Medical School at the University of Texas Health Sciences Center (UTHealth-Houston), Houston, TX 77030, USA; kartik.venkatachalam@uth.tmc.edu; 2 Graduate Programs in Neuroscience and Biochemistry and Cell Biology, MD Anderson Cancer Center and UTHealth Graduate School of Biomedical Sciences, Houston, TX 77030, USA

**Keywords:** longevity, ion channels, lifespan, aging, calcium, ER, lysosomes

## Abstract

Despite significant advances in our understanding of the mechanisms that underlie age-related physiological decline, our ability to translate these insights into actionable strategies to extend human healthspan has been limited. One of the major reasons for the existence of this barrier is that with a few important exceptions, many of the proteins that mediate aging have proven to be undruggable. The argument put forth here is that the amenability of ion channels and transporters to pharmacological manipulation could be leveraged to develop novel therapeutic strategies to combat aging. This review delves into the established roles for ion channels and transporters in the regulation of aging and longevity via their influence on membrane excitability, Ca^2+^ homeostasis, mitochondrial and endolysosomal function, and the transduction of sensory stimuli. The goal is to provide the reader with an understanding of emergent themes, and prompt further investigation into how the activities of ion channels and transporters sculpt the trajectories of cellular and organismal aging.

## 1. Introduction

All biological processes lose their integrity with the passage of time. Aging is the single greatest risk factor for diseases associated with diminished survivorship such as cancer, diabetes, cardiovascular diseases, and neurodegeneration [1,2]. By extension, strategies that ameliorate age-dependent diseases also extend lifespan. Over the past three decades, major advances in gerobiology have been made using model organisms such as *C. elegans* and *Drosophila* [3,4,5,6]. These pioneering studies have established that various biochemical pathways influence the rates of cellular and organismal aging [2]. Emerging insights have informed investigations into the genetics of aging, and prompted the quest to identify drugs that extend healthspan by ameliorating age-related diseases or by slowing the rates of biological aging.

The problem, however, is that barring a few exceptions (e.g., rapamycin and metformin [2]) not many drugs have emerged as suitable candidates to combat aging. Although this barrier has many explanations, the most obvious is the sheer complexity of aging. Age-related loss of physiological integrity stems from a host of defects, including genomic and epigenetic instability, telomere shortening, proteostatic imbalance, chronic inflammation, dysregulated nutrient sensing, mitochondrial defects, cellular senescence, and stem cell exhaustion [1,2]. Most proteins and signaling cascades involved in the regulation of these hallmarks are undruggable.

With perhaps the exception of G-protein coupled receptors, ion channels and transporters count among the most druggable targets in the proteome. This is because domains that mediate ion transport usually interface well with small molecules. Indeed, many naturally occurring biological toxins target ion channels and transporters with exquisite sensitivity, and these entities have been used to engineer novel pharmacological modifiers. Furthermore, since the activity of ion channels and transporters can be assessed in vitro using electrophysiology or optical imaging, it is possible to conduct high-throughput screens to discover suitable activators and inhibitors. Although it might not be possible to extend healthspan or longevity by targeting channels that function in just one pathway, a cogent strategy for the amelioration of age-dependent loss of function could involve the identification and targeting of ion channels and transporters that affect multiple pathways related to organismal aging. With this rationale in mind, this article provides an overview of how aging and longevity are determined by the activities of various ion channels and transporters. The overarching goal of this review is to highlight commonalities and encourage further investigation into the modulation of the activities of ion channels and transporters to influence the rates of cellular and organismal aging.

## 2. Regulation of Aging and Longevity by Ion Channels and Transporters Related to Ca^2+^ Homeostasis and Electrical Excitability

Ca^2+^ is a key second messenger that is involved in the regulation of a host of different pathways, including synaptic transmission, neuronal survival, cellular bioenergetics, and gene expression that range over timescales spanning from milliseconds to hours and days [7,8]. Critical to cellular Ca^2+^ signaling are ATP-consuming transporters that maintain cytosolic Ca^2+^ concentration ([Ca^2+^]) in the nanomolar range by pumping the cation against its steep concentration gradient (~10,000-fold across the plasma membrane and endoplasmic reticulum (ER) membrane) [7,8]. Movement of Ca^2+^ down its concentration gradient involves the opening of Ca^2+^-permeable channels that serve as conduits that permit transient elevations of cytosolic [Ca^2+^], and the attendant activation of relevant cellular pathways [7,9].

**Figure 1:**
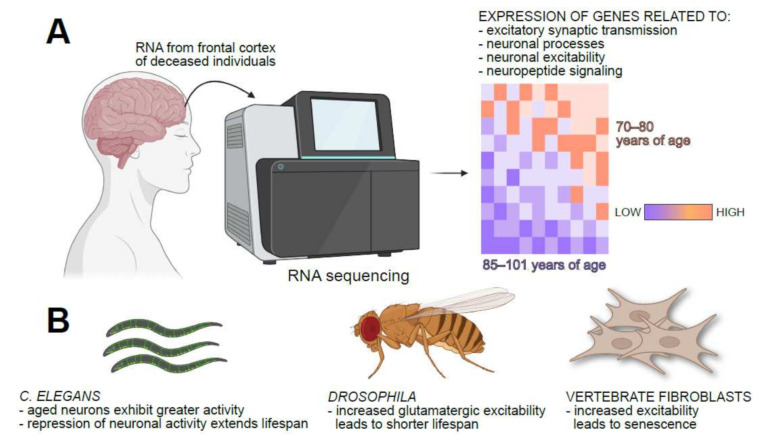
Regulation of cellular and organismal aging by neuronal excitability. (**A**) Sequencing of RNA extracted from the frontal cortices of deceased individuals revealed that expression of genes related to neuronal excitability and excitatory synaptic transmission was relatively lower in long-lived humans (i.e., those who died between the ages of 85–101 years) compared to that in people who died between the ages of 70–80 years. (**B**) In both *C. elegans* and *Drosophila*, neuronal excitability is inversely correlated with longevity, and pharmacological repression of neuronal activity extends lifespan. In non-excitable cells such as fibroblasts, increased electrical excitability is associated with precocious onset of senescence. Images created with BioRender.com (accessed on 20 January 2022).

One of the most exciting recent findings in gerobiology is that expression of genes associated with the maintenance of neuronal Ca^2+^ homeostasis, excitability, and synaptic function is anticorrelated with lifespan in many organisms including humans [10]. Analysis of RNA-seq and microarray datasets generated from frontal cortices isolated immediately post-mortem revealed the downregulation of genes associated with neurotransmission in exceptionally long-lived humans [10] (Figure 1A). Advanced age is also associated with increased neuronal activity in *C. elegans*, and pharmacological attenuation of excitability and its consequences using the Cl^−^ channel agonist, ivermectin, or the L-type voltage-gated Ca^2+^ channel (VGCC) blocker, nimodipine, extended worm lifespan [10] (Figure 1B). Roles for excitability in the regulation of neuronal viability and organismal lifespan are also observed in *Drosophila* [11,12,13,14,15] (Figure 1B). Deletion of the fly K^+^ channel genes, *ether a go-go* (*eag*) and *shaker* (*Sh*)—expected to augment neuronal excitability—led to severe loss of motor coordination, sleep deficits, and shorter lifespan [11,15]. Diminished Na^+^/K^+^ ATPase activity, which leads to a loss of the resting membrane potential and severe neuronal hyperexcitability, also caused shorter lifespan in flies [11,12,16].

The relationship between neuronal excitability and lifespan is conserved in mammals [10]. As is the case in *C. elegans*, ivermectin extends lifespan in a mouse model of amyotrophic lateral sclerosis (ALS) [17]. Plasma membrane excitability is also an important determinant of oncogene-induced senescence (OIS) in non-excitable mammalian cells [18] (Figure 1B). A genetic screen revealed that loss of *SCN9A*, the gene encoding a voltage-gated Na^+^ channel, protected human fibroblasts from OIS [18]. Oncogenic signals or activation of the tumor suppressor, p53, led to the induction of *SCN9A* with NF-kB as an intermediary [18]. Elevations in SCN9A abundance, which is expected to augment plasma membrane excitability, led to senescence via the retinoblastoma (Rb) pathway [18]. Whether NF-kB signaling, p53, or Rb also contribute to the shorter lifespan observed in animals with chronic neuronal depolarization (see below) remains to be addressed.

### 2.1. Regulation of Aging by Ca^2+^ Channels in the Plasma Membrane

Converging lines of evidence indicate a central role for plasma membrane-resident Ca^2+^ channels in the regulation of longevity [19,20]. Aged mammalian nervous system is characterized by increased abundance of L-type VGCCs [21]. Concordantly, verapamil-mediated inhibition of L-type channels ameliorated neurodegeneration and extended lifespan in a mouse model of ALS [22]. Similarly, N-type VGCCs (Cav2.2) contribute to age-related neuroinflammation [23]. Pharmacological inhibition of Cav2.2 channels blunts neuroinflammation in aged mice [23]. In endothelial cells, VGCC activity contributes to replicative senescence, and the VGCC inhibitor, nifedipine, reduces the fraction of senescent cells in the endothelium [24]. These findings in mammalian models agree with those in aged *C. elegans* as pertaining to the extension of lifespan conferred by the administration of L-type VGCC inhibitors, nimodipine, and verapamil [10,19]. In further agreement, knockdown of the VGCC subunit, *unc-36*, extends *C. elegans* lifespan [20].

Members of the transient receptor potential (TRP) superfamily of cation channels are also important in age-dependent changes in various cell types. Loss of the gene encoding TRPC5 protects mouse endothelial cells from the induction of senescence markers such as p53 and p21, and from oxidative stress-induced senescence [25]. These findings point to TRPC5 as an actionable target in the regulation of vascular aging. Activity of members of the Melastatin subfamily of TRP channels (TRPM) also impacts the onset of cellular senescence. TRPM2 conductance is positively correlated with oxidative stress-induced neuronal senescence [26]. Indeed, the mitigation of oxidative stress by glutathione is associated with the inhibition of TRPM2, whereas depletion of glutathione is associated with augmented TRPM2 activity [26]. In contrast, TRPM7 and TRPM8 abundances are elevated in pancreatic cancer where they play roles in the amelioration of OIS and replicative senescence [27,28,29]. Knockdown of the genes encoding TRPM7 and TRPM8 induces senescence in pancreatic cancer cells [27,28,29].

Years of study have pointed to a critical role for NMDA receptors in Ca^2+^ excitotoxicity [30]. As such, hyperactive N-methyl D-aspartate (NMDA) receptors contribute to the shortening of lifespan observed in models of neurodegenerative disease. For instance, spinocerebellar ataxia type 1 (SCA1), which is caused by a CAG repeat expansion in the *SCA1* gene, is associated with the pathologically elevated activity of extrasynaptic NMDA receptors [31]. Long-term administration of the NMDA receptor antagonist, memantine, attenuated cerebellar neurodegeneration and extended animal lifespan in a mouse model of SCA1 [31]. Similarly, in a *Drosophila* model of *C9ORF72* ALS, inhibition of NMDA receptors delayed motor dysfunction and extended animal lifespan [32]. These studies demonstrate that excessive NMDA receptor activity in models of neurodegenerative diseases underlies the loss of neuronal viability and shortening of lifespan.

On the other hand, loss of cognitive function associated with advanced age may stem from an age-dependent decrease in NMDA function. NMDA receptor densities in parts of the brain such as the hippocampus decrease with age, and in models of Alzheimer’s disease [33,34,35]. These changes underlie aspects of age-dependent cognitive decline. In this context, NMDA receptor activity has been linked to a transcriptional antioxidant response aimed at preventing activity-dependent loss of synaptic transmission [36]. Interventions that promote NMDA receptor function in aged animals, therefore, ameliorate the onset of learning and memory deficits [34,35,37,38,39]. Together, these studies point to the importance of optimal NMDA receptor activity for the maintenance of brain resilience with the passage of time, such that either an increase or decrease in NMDA receptor activity can shorten lifespan.

### 2.2. Regulation of Aging and Longevity by Ca^2+^ Channels in the Endoplasmic Reticulum (ER)

**Figure 2:**
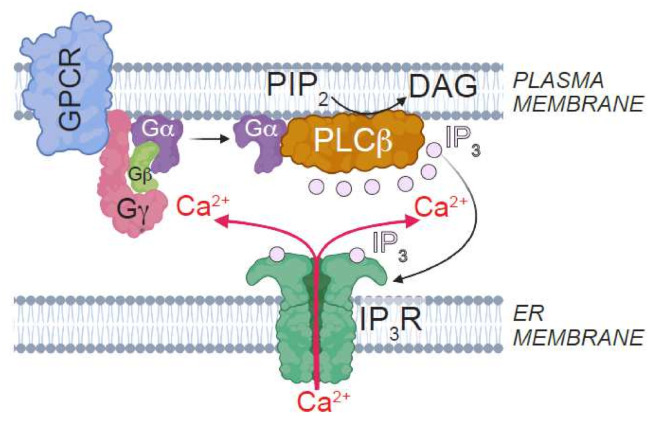
The PLCβ–IP_3_R signaling axis. Stimulation of Gαq-coupled GPCRs leads to the activation of PLCβ. PLCβ hydrolyzes the membrane phosphoinositide, PIP_2_, to generate the second messengers, diacylglycerol (DAG) and IP_3_. While DAG is retained in the plasma membrane, IP_3_ diffuses through the cytosol and activates IP_3_Rs, which are localized to the ER membrane. Activation of IP_3_Rs results in the release of ER Ca^2+^. Image created with BioRender.com (accessed on 20 January 2022).

The notion that deviation from the optimal range of channel activity can become toxic is also the case for Ca^2+^ channels that localize to the ER [8]. Inositol trisphosphate receptors (IP_3_Rs) are ER-resident Ca^2+^ channels that are activated by IP_3_ generated by phospholipase C isoforms (PLCβ) such as those coupled to G-protein coupled receptors (GPCRs) [40] (Figure 2). In case of the gene encoding IP_3_R type 1 (IP_3_R1, encoded by *ITPR1*), loss-of-function mutations result in spinocerebellar ataxia types 15 and 29 (SCA15 and SCA29, respectively), Gillespie syndrome, and sporadic infantile-onset cerebellar ataxia [41,42,43,44,45,46]. While most mice carrying *Itpr1* deletion die as embryos, the escapers exhibit severe ataxia and tonic-clonic seizures that lead to death by the weaning period [47]. Furthermore, mutations that lowered the abundance of IP_3_R1 in the cerebellar Purkinje neurons induced ataxia, and sensitized neurons to cell death during ER stress [41,48]. On the other hand, elevated IP_3_R1 activity underlies the neuropathology in SCA2 and SCA3 [49,50]. Augmented IP_3_R1 activity also contributes to neuronal cell death in Huntington disease (HD) and Niemann-Pick C1 disease [51,52]. Therefore, either an increase or decrease in IP_3_R1 activity is toxic to neurons, and can thus shorten lifespan.

Neurons are also sensitive to the dosage of the IP_3_R2 isoform. Suggesting that IP_3_R2 protects against ALS, deletion of *Itpr2* in the SOD1^G93A^ mouse model of ALS promoted inflammation and diminished animal survival [53]. Therefore, higher *ITPR2* expression in the spinal cord of humans with ALS [53], suggests the engagement of a protective response involving IP_3_R2. In the absence of the ALS-related mutations, however, IP_3_R2 contributes to the adverse consequences of advanced age. *Itpr2* knockout mice exhibit decreased age-related pathology resulting in longer lifespan, resistance to metabolic stress, and diminished immunosenescence [54]. Even at the cellular level, deletion or repression of *Itpr2* prevented OIS or ROS-induced senescence [54,55,56,57]. This effect on senescence is due to diminished interorganellar transfer of Ca^2+^ between the ER and mitochondria, which is otherwise mediated very effectively by IP_3_R2.

In *Drosophila*, *itpr* overexpression in glutamatergic neurons is sufficient to shorten animal lifespan [12]. Knockdown of *itpr* mitigates the effects of neurodegeneration-causing transgenes on fly lifespan and locomotion [12,58]. Together, these findings point to a necessary and sufficient role for neuronal IP_3_R activity in the regulation of fly lifespan. Several lines of evidence suggest that elevated IP_3_R activity was a consequence of chronic depolarization of fly neurons expressing neurodegeneration-causing transgenes [12]. First, expression of ALS and tauopathy-related transgenes in fly glutamatergic neurons led to concomitant loss of membrane potential and elevated IP_3_R-mediated ER Ca^2+^ release. Second, abbreviated lifespans in flies expressing these transgenes were ameliorated by the knockdown of *itpr*. Third, expression of dominant-negative variant of a subunit of the Na^+^/K^+^ ATPase to prevent neuronal repolarization shortened animal lifespan via IP_3_R activity. Fourth, Ca^2+^ imaging experiments demonstrated that depolarization was sufficient to induce greater IP_3_R-dependent ER Ca^2+^ release in response to invariant concentrations of agonists of PLCβ-coupled receptors.

**Figure 3:**
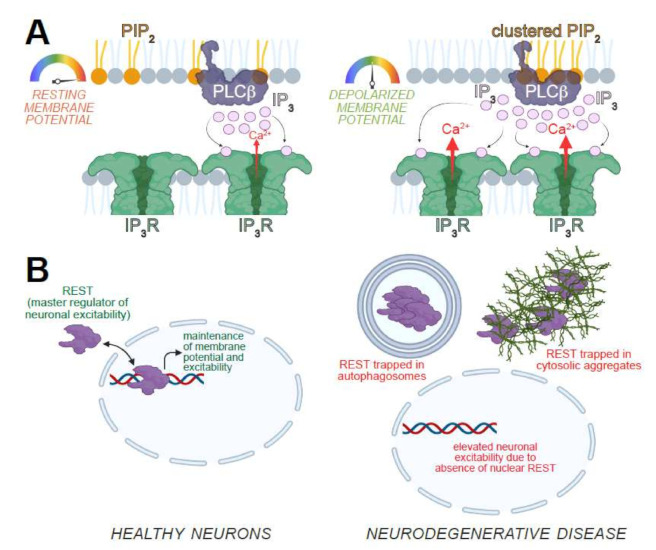
Consequences and regulators of neuronal excitability. (**A**) Left, at resting membrane potentials, PLCβ hydrolyzes the PIP_2_ moieties associated with the enzyme. Right, plasma membrane depolarization results in enhanced PIP_2_–PLCβ clustering. Subsequent, stimulation of PLCβ-coupled receptors in depolarized neurons results in the augmentation of PIP_2_ hydrolysis, IP_3_ production, and IP_3_R-dependent ER Ca^2+^ release. (**B**) Left, REST is a master regulator of membrane excitability in healthy neurons. Nuclear translocation of REST allows for the repression of RE1-containing gene such as those encoding channels and pumps. Repression of these genes is necessary for the proper maintenance of membrane potential and excitability. Right, in neurodegenerative disease, REST is sequestered away from the nucleus resulting in derepression of target genes and the augmentation of excitability and genes related to synaptic transmission. Images created with BioRender.com (accessed 25 January 2022).

Investigation of the mechanisms by which plasma membrane potential modulates IP_3_R activity revealed that depolarization induced greater association between PLCβ and its phosphoinositide substrate, phosphatidylinositol-(4,5)-bisphosphate (PIP_2_). Therefore, stimulation of PLCβ-coupled receptors in depolarized neurons enhanced PIP_2_ hydrolysis, which resulted in higher levels of IP_3_ and augmented IP_3_R activity [12] (Figure 3A). Either the deletion of the gene encoding a fly PLCβ isoform or the knockdown of the gene encoding a PLCβ-coupled GPCR, prevented depolarization from abbreviating fly lifespan [12]. Even in *C. elegans*, attenuation of PLCβ signaling extended lifespan by repressing the insulin–DAF16/FOXO axis [59,60,61].

### 2.3. Transcriptional Control of Excitability and Longevity

The relationship between longevity and the neuronal excitability is mediated by Repressor Element-1 (RE1) Silencing Transcription Factor (REST, also called NRSF), which represses genes carrying the RE1 motif [10,62,63]. REST appears to promote resilience to age by decreasing the expression of genes necessary for promoting neuronal excitability [10,64] (Figure 3B). REST-deficient mice exhibit cortical hyperexcitability, a propensity for epileptic seizures, and enhanced mortality [10]. In *C. elegans*, knockdown of REST orthologs augments neuronal excitability and shortens lifespan, whereas elevated REST activity represses neuronal excitability and lengthens animal lifespan [10]. In worms, REST and attendant neuronal excitability impact on longevity via the regulation of DAF16/FOXO signaling [10,59,60].

The findings that expression of neurodegeneration-causing transgenes in *Drosophila* glutamatergic neurons results in both hyperexcitability and shorter lifespan [12] is consistent with the notion of REST dysregulation in models of neurodegeneration [65]. Deletion of REST is sufficient to induce degeneration in mouse and *C. elegans* nervous systems [66]. REST is neuroprotective in aged human neurons, iPSC-derived neurons in patients with Alzheimer’s disease (AD), and in mouse models of AD [10,66,67]. Mechanistically, REST function is lost in AD patients and in individuals with age-dependent cognitive impairment due to increased sequestration into autophagosomes, and depletion from the nucleus [66,67] (Figure 3B). It is also possible that REST might get trapped in protein aggregates that are observed in many neurodegenerative diseases (Figure 3B), although this possibility has not been demonstrated directly. An exception to the model that REST is ubiquitously neuroprotective is the case in HD. In HD neurons, REST exhibits aberrantly high nuclear localization, increased RE1 occupancy, and expected gene repression [68,69]. Whether these outcomes contribute to HD pathology, and how this phenotype relates to that observed in AD remain incompletely understood.

## 3. Regulation of Aging and Longevity by Mitochondrial Ion Channels and Transporters

Mitochondria are critical for life at both cellular and organismal levels. Mitochondrial function declines in older animals, and the underlying mechanisms are major contributors to the adverse outcomes of advanced age [70]. This section aspires to describe the contributions of mitochondrial ion channels and transporters to the regulation of aging and longevity.

### 3.1. Mitochondrial Ca^2+^ Uniporter

**Figure 4:**
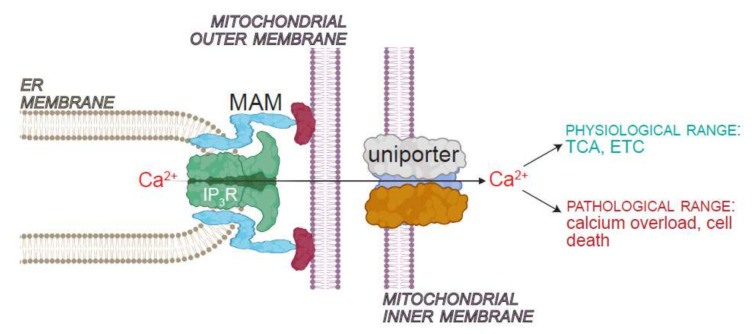
Interorganellar Ca^2+^ transfer between ER and mitochondria. ER and mitochondrial outer membrane are physically tethered at sites called mitochondria associated membranes (MAMs). MAMs constitute the regions of metabolite exchange between the two organelles. A fraction of IP_3_Rs localize to MAMs, where they mediate the transfer of Ca^2+^ from the ER lumen to the perimitochondrial region. The mitochondrial Ca^2+^ uniporter resides in the inner mitochondrial membrane, and is responsible for the transfer of Ca^2+^ into the mitochondrial matrix. Physiological Ca^2+^ elevations in the matrix are necessary for mitochondrial bioenergetics and the production of ATP via TCA and ETC. Ca^2+^ overload, however, results in pathology and eventual cell death. Image created with BioRender.com (accessed 24 January 2022).

Uptake of Ca^2+^ into the mitochondrial matrix is critical for many aspects of mitochondrial function, including ATP production (Figure 4). Ca^2+^ transport across the inner mitochondrial membrane is mediated by the mitochondrial Ca^2+^ uniporter (MCU) and its regulators, mitochondrial Ca^2+^ uptake 1-3 (MICU1-3), mitochondrial Ca^2+^ uniporter regulator 1 (MCUR1), and essential MCU regulator (EMRE) [71,72,73,74,75]. Close coupling between the ER and mitochondria permits the Ca^2+^ ions released via IP_3_Rs to be taken up into the mitochondrial matrix through the uniporter [71,76] (Figure 4). Coordinated activities of IP_3_Rs and MCU, therefore, permit Ca^2+^ transport from the ER to mitochondria, which in turn, stimulates mitochondrial bioenergetics (Figure 4). This mode of interorganellar Ca^2+^ transport is needed for *C. elegans* longevity [61]. In agreement, knockdown of *MCU* in *Drosophila* neurons further abbreviates the lifespan of animals experiencing precocious mortality due to diminished ER–mitochondria Ca^2+^ exchange [12]. In mice, MICU3 abundance and MCU-dependent mitochondrial Ca^2+^ uptake decrease in the aging skeletal muscle leading to sarcopenia [77]. Restoration of MICU3 abundance in mouse muscle increased myogenesis and delayed sarcopenia [77].

While uniporter activity within the physiological range is necessary for animal viability, transporter overactivity has been observed in many pathological conditions (Figure 4). MCU and MICU1 contribute mitochondrial Ca^2+^ overload that occurs in various neurodegenerative and pathological conditions that shorten lifespan. Untrammeled MCU activity contribute to neurodegeneration in SCA, and knockdown of *Mcu* in mouse neurons ameliorates mitochondrial Ca^2+^ overload and excitotoxicity stemming from NMDA receptor activation [78,79]. In mouse cardiomyocytes, deletion of *Mcu* protects from ischemia reperfusion injury [80,81]. Furthermore, knockdown of the gene encoding a regulatory partner of MCU, MCUR1, attenuates mitochondrial Ca^2+^ uptake and ATP production, and drives pro-survival pathways such as AMPK activation and autophagy [75].

MCU also senses and responds to mitochondrial oxidative stress and redox balance. Glutathione moieties can be added to MCU, which attenuates the Ca^2+^ transport activity of the protein, and thereby, limits mitochondrial metabolism and reactive oxygen species (ROS) production [82]. In *Drosophila* muscle, MCU is necessary for oxidative stress-induced cell death, and deletion of fly *MCU* confers robust resistance to oxidative stress-dependent lethality [83]. Even OIS requires MCU-dependent mitochondrial Ca^2+^ overload subsequent to ER Ca^2+^ release via IP_3_Rs [56]. In view of the model that ROS production contributes to aging [84,85], MCU could be considered as drivers of aging and senescence.

### 3.2. Other Mitochondrial Channels and Transporters

Opposing the effects of the uniporter are transporters that extrude matrix Ca^2+^. Removal of matrix Ca^2+^ under physiological conditions is largely dependent on the mitochondrial Na^+^/Ca^2+^/Li^+^ exchanger (NCLX), with the permeability transition pore playing a role during Ca^2+^ overload [86,87,88,89]. Although Ca^2+^/H^+^ exchange has also been proposed to serve a role in the removal of mitochondrial Ca^2+^, molecular identity of the exchanger remains controversial [89,90,91].

Deletion of NCLX in the adult mouse heart has led to sudden death due to heart failure [88]. The underlying mechanism, not surprisingly, involved mitochondrial Ca^2+^ overload, unremitting ROS production, and cell death [88]. Conversely, increased abundance of NCLX in the mouse heart protects against ischemic cell death and heart failure [88]. In dissociated hippocampal neurons challenged with excitotoxic stimuli, knockdown of the gene encoding NCLX further dysregulates mitochondrial Ca^2+^ homeostasis and promotes ROS production [92]. Diminished abundance of NCLX in neurons and glia, and attendant impairments in the removal of mitochondrial Ca^2+^ are sufficient to elicit neurodegeneration [92]. Indeed, deletion of NCLX accelerates amyloid plaque formation, tau neuropathology, and the rate of memory decline in a mouse model of AD [93]. Mitochondrial [Ca^2+^] is also constitutively elevated due to diminished NCLX activity in multiple mouse models of Parkinson’s disease [94,95,96].

Many studies have pointed to roles for K^+^ channels in mitochondrial function. Small conductance Ca^2+^-activated K^+^ channels (SK2 channels) localize to the inner mitochondrial membrane and mediate mitochondrial K^+^ currents [97]. Activation of mitochondrial SK2 channels protects cells against mitochondria-dependent cell death when challenged by excitotoxic insults [97]. In *C. elegans*, pharmacological activation of SK channels promotes longevity by conferring resistance to ferroptosis [98]. Big conductance Ca^2+^-activated K^+^ channels (BK channels) are also found in the inner mitochondrial membrane of cardiomyocytes, where they mediate Ca^2+^-activated K^+^ currents and protect the heart from ischemic injury [99]. In *Drosophila*, absence of mitochondrial BK channels led to increased ROS production and abbreviated fly lifespan, whereas overexpression of these channels led to long-lived animals [100].

## 4. Mitochondrial Uncoupling Proteins and Longevity

**Figure 5:**
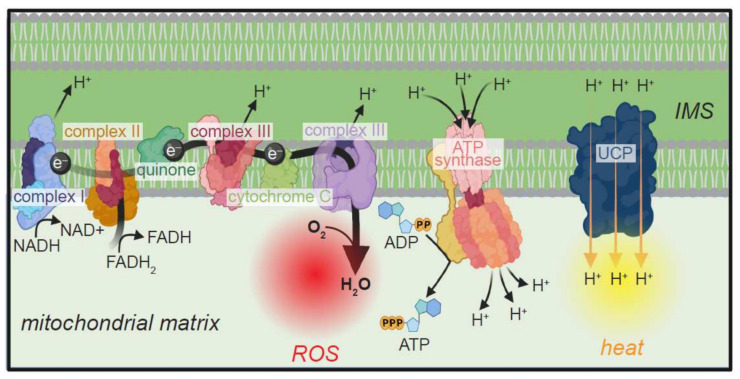
Uncoupling protein (UCP) mediated dissipation of the mitochondrial proton (H^+^) gradient. ETC involves the transfers of electrons (e^−^) from reducing equivalents (NADH and FADH_2_) to molecular oxygen (O_2_). e^−^ transport is coupled with the movement of H^+^ from the mitochondrial matrix to the intermembrane space (IMS). Subsequent dissipation of the electrochemical gradient through ATP synthase drives the production of ATP. Transfer of e^−^ to O_2_ also leads to the generation of superoxide ions and ROS. By mediating the transport of H^+^ down their electrochemical gradient, UCPs dissipate the protonmotive force. Loss of protonmotive force results in the production of heat and prevents ATP production. Upon UCP-mediated H^+^ leak, diminished transfer of e^−^ to O_2_ mitigates ROS production. Image created with BioRender.com (accessed 24 January 2022).

Uncoupling proteins (UCPs) are integral proteins of the inner mitochondrial membrane that mediate the transport of protons from the intermembrane space (IMS) to the matrix [101,102] (Figure 5). As such, mitochondrial UCPs dissipate the electrochemical gradient across the inner mitochondrial membrane, which otherwise drives ATP synthesis via ATP synthase [101] (Figure 5). Because dissipation of the protonmotive force leads to release of the energy derived from oxidized substrates in the form of heat, UCP1-mediated proton leak drives thermogenesis in brown adipose tissue [101,102,103,104,105]. UCPs 2-5, on the other hand, have limited roles in thermogenesis, and are needed for the attenuation of mitochondrial oxidative stress, regulation of cellular and organismal metabolism, and antimicrobial immunity [101,106,107,108,109,110,111,112].

The mechanisms of UCP-dependent proton transport have been studied extensively in the context of UCP1. These studies have revealed that UCPs are dimers of 6 transmembrane domain-containing proteins [102,113]. At the core of the dimer is a hydrophilic pore [102]. UCP-mediated proton transport requires free fatty acids, which are purported to function via one of two possible mechanisms [101,114]. The “proton buffering model” argues that fatty acids transfer protons to proton-buffering amino acids in the pore, which then shuttle the ions across the membrane [101]. Alternatively, the “fatty acid cycling model” contends that fatty acid anions accept protons in the intermembrane space and transport protons across the membrane by directly traversing the pore [101].

### Role of UCP Proteins in the Response to Oxidative Stress

ROS—well-established byproducts of mitochondrial metabolism—can damage DNA, proteins, and lipids, and are therefore drivers of senescence. Because the mitochondrial protonmotive force is positively correlated with superoxide production [101,115], activation of UCP2 and UCP3 mitigates cellular oxidative stress, and thereby, counters the onset of senescence [101,102,107,108,109,110]. Conversely, loss of either UCP2 or UCP3 is associated with increased levels of mitochondrial ROS [109,110,116,117].

There are clearly some benefits associated with increased ROS in the absence of UCPs. Elevated ROS production in macrophages isolated from *Ucp2^−/−^* mice greatly restricts growth of *Toxoplasma gondii*, making the animals resistant to death from *Toxoplasma* infection [109]. More generally, however, ROS promotes senescence and aging [84,85,102,118,119,120,121,122]. Therefore, higher levels of proton leak via UCP2 or UCP3, which mitigate oxidative stress, are correlated with longer lifespans in mice [123,124]. Ectopic overexpression of human *UCP2* or *UCP3* in fly and/or mouse neurons is sufficient to prolong the animals’ lifespans, and confer greater resistance to ROS [120,125,126,127]. These beneficial effects depend on the induction of pro-longevity genes in hypothalamic neurons of mice, or in insulin-producing fly neurons, which are reminiscent of hypothalamic neurons [125,126,127]. Given the role for the hypothalamus in the regulation of feeding and energy homeostasis, these findings raise the intriguing possibility that UCP activity regulates lifespan by influencing the animals’ feeding behavior. Indeed, caloric restriction in mice—a reliable pro-longevity factor—augments the expression of *Ucp2* and *Ucp3*, promotes mitochondrial proton leak, and lowers ROS production [128,129]. The convergence of multiple longevity-related pathways on UCP2 and UCP3 speaks to the pro-survival roles of uncoupling, and supports the “uncoupling to survive” model [123,130].

Although the predominant role for UCP1 is thermogenesis in brown adipose tissue, abundance of this protein does correlate with lower incidence of age-related disease [131]. In humans, polymorphisms in the regulatory regions of *UCP1* that result in greater expression of the gene are associated with improved longevity [132]. Overexpression of *Ucp1* in mice is sufficient to extend lifespan by lowering the incidence of cancer and atherosclerotic lesions, and by correcting preexisting metabolic dysfunction [131]. Conversely, *Ucp1^−/−^* mice exhibit greater susceptibility to obesity at advanced age when reared on a high-fat diet (HFD) [133]. Given the adverse effects of obesity on lifespan, these findings point to a role for UCP1 in mitigating the consequences of unhealthy diet on age-related decline of healthspan.

## 5. Regulation of Lifespan by Ion Channels Involved in Autophagy and Lysosomal Proteostasis

Autophagy and lysosomal protein degradation constitute major axes of proteostasis in metazoans. By coordinating the removal of toxic macromolecules and damaged organelles, these processes counteract the stochastic accumulation of cellular debris that otherwise promote aging [134,135,136]. Therefore, hypermethylation of autophagy and lysosomal genes tends to diminish protein degradation in aged organisms [137], whereas upregulation of autophagy and lysosomal function improves proteostasis and extends healthy lifespan [134]. Induction of autophagy is sufficient to confer resistance to oxidative stress and insulin sensitivity in mice reared on a high-fat diet, and ameliorate the toxic consequences of polyglutamine expansion in mouse and fly models [138,139]. Ubiquitous overexpression of *Atg5*, a gene that encodes a protein needed for autophagosome formation, extends mouse lifespan via the augmentation of leanness, insulin-sensitivity, and oxidative stress tolerance [140]. Even the extension of lifespan brought about by diminished insulin-signaling in *Drosophila* or dietary restriction in *C. elegans* are dependent on autophagy and lysosomal activity [141,142,143]. Genetic upregulation of chaperone-mediated autophagy (CMA), which involves LAMP-2A-dependent direct lysosomal targeting of select proteins, also enhances proteostasis, mitigates oxidative stress, and preserves organ function in aged mammals [144,145]. Conversely, the repression of autophagy by deletion of *Atg5* or *Atg7* in mice and flies promotes aging, neurodegeneration, and shorter lifespan via dysregulation of proteostasis and mitochondrial function [146,147,148,149,150,151].

### 5.1. Involvement of Vesicular Ion Channels in Endolysosomal Function and Lifespan

Autophagic protein degradation is comprised of a series of vesicular trafficking events that originate with the sequestration of cargo into autophagosomes. These double membrane-bound organelles then fuse with endolysosomal vesicles in order to permit hydrolytic enzymes to gain access to material destined for degradation [152]. In *Drosophila*, hybrid organelles formed upon the fusion of late-endosomes and autophagosomes (amphisomes) fuse with lysosomes in a Ca^2+^-dependent process requiring the endolysosomal cation channel, TRPML [153,154]. Loss of *trpml*, therefore, prevents the fusion of amphisomes with lysosomes, which leads to the accumulation of amphisomes and diminished endolysosomal degradation [153,154]. These phenotypes also characterize Mucolipidosis type IV (MLIV)—a lysosomal storage disease that stems from the loss of *MCOLN1*, the gene encoding the human ortholog [155,156,157,158].

**Figure 6:**
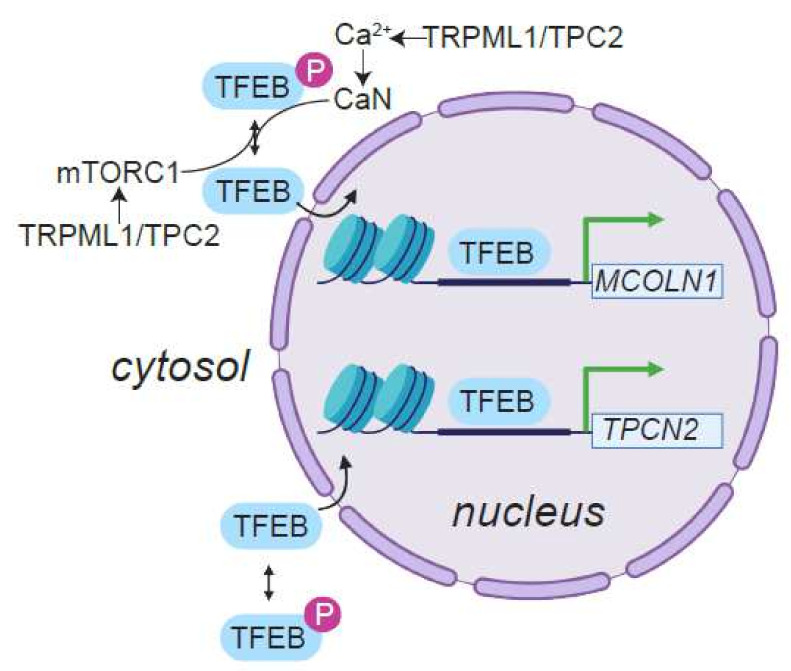
Transcriptional regulation of genes encoding endolysosomal ion channels. Within the cell nucleus, TFEB, and related transcription factors such as TFE3 and MITF, bind to the promoters of genes that encode autophagy and endolysosomal proteins. Figure shows dephosphorylated TFEB bound to the promoters of *MCOLN1* and *TPCN2*, which encode TRPML1 and TPC2, respectively. Phosphorylated TFEB is retained in the cytosol leading to diminished expression of endolysosomal genes. TRPML1 and TPC2 are endolysosomal cation channels that regulate nucleocytoplasmic translocation of TFEB activity in different ways. By driving autophagic and endolysosomal flux, TRPML1 and TPC2 are needed for mTORC1 activation, which in turn, phosphorylates TFEB leading to retention of the transcription factor in the cytosol. On the other hand, release of endolysosomal Ca^2+^ via TRPML1 and TPC2 activates calcineurin (CaN), which dephosphorylates TFEB, and thereby, promotes the translocation of the transcription factor into the nucleus. Image created with BioRender.com (accessed 24 January 2022).

In flies, diminished amphisome–lysosome fusion in the absence of functional TRPML leads to decreased production of amino acids that are generated from endolysosomal protein degradation [153,154]. Given the roles for endolysosomal amino acids in the activation of the mTORC1 kinase complex [159], loss of TRPML results in diminished mTORC1 activation—a phenotype that can be suppressed by dietary administration of a high-protein diet [153,154]. The role for TRPML1 in mTORC1 activation is also conserved in mammals [160,161,162] (Figure 6). Interestingly, the inverse relationship between mTORC1 activity and endolysosome/autophagosome biogenesis (via TFEB, see below) ensures that loss of TRPML results in upregulation of endolysosomal biogenesis, which may explain the accumulation of lysosomes in MLIV [153,163,164,165].

Based on the aforementioned insights, one can appreciate that the regulation of aging and lifespan by the TRPML family of endolysosomal ion channels is complex. On the one hand, loss of TRPMLs leads to the accumulation of undigested endosomal material, whereas their transcriptional upregulation promotes the exocytosis and clearance of toxic macromolecules [166]. In this regard, TRPMLs are necessary for proteostasis. Indeed, the loss of mouse TRPML1 or fly TRPML leads to proteostatic imbalance, severe loss of neuronal function, and abbreviated lifespans [167,168]. It is worth noting that besides inhibiting the mTORC1 complex, the FDA-approved, longevity-promoting drug, rapamycin [169] also activates TRPML1 independently of mTORC1 [170]. Consequently, rapamycin-induced autophagic flux is attenuated in TRPML1-deficient cells [170]. It is, therefore, possible that some of the pro-longevity effects of rapamycin stem from the activation of TRPML1 and the attendant improvement of cellular proteostasis.

On the other hand, since decreased mTORC1 activity is associated with the extension of lifespan in a variety of organisms [171,172,173,174,175], attenuation of mTORC1 activity in the absence of TRPMLs also have beneficial consequences. In agreement, abbreviation of lifespan upon the expression of neurodegeneration-causing transgenes in fly neurons is suppressed by the concomitant knockdown of *trpml* [12]. Additionally, TRPML1 contributes to the toxicity in a presenilin knockout mouse model of Alzheimer’s disease [176]. Delineating the relative roles of TRPMLs in the regulation of lifespan under physiologically normal or pathological conditions would likely require further investigation.

Two-pore channels (TPCs) are a class of endolysosomal cation channels that orchestrate vesicular trafficking events by releasing vesicular Ca^2+^ and Na^+^ in response to NAADP and PI(3,5)P_2_ [177,178,179,180,181,182,183,184]. TPC function is of relevance to autophagy, mTORC1 activity, protein homeostasis, and cholesterol homeostasis [185,186,187,188] (Figure 6). Not surprisingly, deletion of the gene encoding TPC2 in mice results in skeletal muscle atrophy due to defective lysosomal proteolysis, accumulation of undigested autophagic vacuoles, and heightened sensitivity to starvation [186]. Similar roles for TPCs in ensuring autophagic and endolysosomal flux has also been reported in cardiac muscle [185]. Elevated TPC2 activity is observed in cells expressing Parkinson’s disease associated mutations in LRRK2 [189], suggesting that attenuation of TPC2 activity (as is the case of TRPML1) might be beneficial in late-onset neurodegenerative diseases.

Cl^−^ represents the major endolysosomal counter ion. ClC-6 and ClC-7 and endolysosomal chloride transporters are required for normal lysosomal function [190,191,192]. Loss of these transporters result in a variety of neuropathological alterations, including lysosomal storage, progressive neuron loss, and microglial activation [193,194,195]. The progressive nature of these phenotypes suggests a role for these proteins in the maintenance of neuronal function and survival at advanced age.

Critical to the regulation of amino acid availability and mTORC1 activity are vesicular solute carriers belonging to the SLC family of transporters. Among the many amino acid transporters critical for mTORC1 activation is the lysosomal amino acid transporter, SLC38A9 [196,197,198]. Loss of SLC38A9 decouples mTORC1 from amino acids such as arginine, whereas overexpression of *SLC38A9* results in the constitutive activation of mTORC1, even in the absence of amino acids [196,197,198]. Therefore, SLC38A9 is not only a vesicular transporter needed for the efflux of amino acids from the lysosome, but is also an amino acid sensor that synchronizes mTORC1 activity to amino acid abundance [196,197,198,199]. Besides serving as a sensor and transporter for amino acids, SLC38A9 also harbors cholesterol-binding motifs [200]. Association of cholesterol with SLC38A9 activates mTORC1 independently of the transporter’s role in endolysosomal amino acid efflux [200]. Other amino acid transporters linked to the regulation of mTORC1 include SLC38A5, SLC7A5 (also called LAT1) and the glutamine transporter SLC1A5 (also called ASCT2) [201,202,203,204,205]. Consistent with the roles for mTORC1 in aging [171,172,173,174,175], emerging evidence suggests that modulation of the activity of amino acid sensors, such as the ones described here, could be an effective strategy to influence rates of aging [206]. Indeed, polymorphisms in genes encoding amino acid transporters are associated with age-related changes in physical performance (e.g., grip strength and walking speed) [207]. Conversely, there is increased abundance of amino acid transporters such as SLC7A5, SLC1A5, and SLC38A5 in inflammation and cancer [204,205,206,208]. Together, these studies point to critical roles for amino acid transporters in the regulation of aging and longevity.

### 5.2. Transcriptional Regulation of Endolysosomal Function

A major advancement in the field of lysosomal biology was the identification of transcription factors (TFEB, TFE3, and MITF) that function as master regulators of autophagy and endolysosomal biogenesis [163,164,165,209,210,211,212,213,214]. Genetic or pharmacological activation of these transcription factors promotes the clearance of cellular debris and aggregate-prone toxic macromolecules [166,215,216,217]. As such, TFEB maintains the quiescent state of neural stems cells, and has been positively correlated with longevity in *C. elegans* and mice [218,219,220,221,222,223,224]. TFEB and TFE3 are needed for the maintenance of whole-body metabolism in response to changes in various environmental stimuli including diet [225]. Dietary restriction—an established mode of lifespan extension—promotes TFEB-dependent gene expression in mouse hepatocytes [222].

Many genes that encode endolysosomal ion channels, including TRPML1 and TPC2, are under the control of TFEB [211,212,226,227] (Figure 6). In turn, nuclear translocation of TFEB, which is dependent on the phosphorylation status of the protein, is regulated by TRPML1 and TPC2 (Figure 6). On the one hand, TRPML1- and TPC2-dependent activation of mTORC1 ensures TFEB phosphorylation and cytosolic retention [160,161,162,163,164] (Figure 6). These findings explain why the lack of TRPML1, results in nuclear translocation of TFEB and unremitting endolysosomal biogenesis. On the other hand, endolysosomal Ca^2+^ release can also dephosphorylate and promote nuclear translocation of TFEB via the protein phosphatase, calcineurin (CaN) [228] (Figure 6). This model portends a feed-forward cycle, whereby TRPML1 and TPC2 activation, which are under TFEB transcriptional control, promotes further TFEB-dependent endolysosomal biogenesis.

While TFEB-driven autophagy and endolysosomal biogenesis delays aging via the clearance of toxic macromolecules, unremitting activation of TFEB can paradoxically shorten lifespan [141]. Either the overexpression and/or constitutive nuclear localization of TFEB and TFE3 promote the growth of various types of cancer [226,227,229,230,231,232,233,234,235,236,237,238,239,240]. Additionally, amplification of the *MITF* locus and functional upregulation of MITF are potent drivers of melanoma [213,241,242,243,244,245,246]. These findings point to the importance of maintaining autophagy and endolysosomal function at an optimal level because deviation from this optimum in either direction has deleterious consequences to healthy lifespan. In cancers driven by TFEB/TFE3/MITF, expression of genes encoding endolysosomal cation channels, TRPML1, TRPML2 and TPC2, is elevated, and either the knockdown or pharmacological inhibition of these channels attenuates the growth and invasiveness of the tumors [160,226,227,247,248,249,250,251,252]. Taken together, these studies point to the highly context-dependent relationship between endolysosomal ion channels and healthspan.

## 6. Regulation of Ion Channel Activity by the Longevity Factor, Klotho

Mutations in the mouse *klotho* gene results in animals that develop normally, but prematurely exhibit several features of accelerated aging including neurodegeneration, atherosclerosis, osteoporosis, infertility, atrophy of the skin and other organs, and shorter lifespan [253,254]. Conversely, overexpression of *klotho* results in a 20–30% extension of mouse lifespan via the repression of insulin signaling and the amelioration of oxidative stress [255,256]. Overexpressed *klotho* also enhances cognition by the enhancement of synaptic plasticity [257]. These roles for Klotho in healthy aging are also observed in humans [258,259,260].

The product of the *klotho* gene is a type-I single pass transmembrane protein that localizes to the cell surface [256]. The extracellular domain of the protein exhibits homology to glycosidases/sialidases that can cleave the b-glycosidic linkage in sugars, glycoproteins and glycolipids, and remove sialic acid residues from membrane proteins [253,256,261,262,263]. Although expression of *klotho* is highest in the kidneys, the extracellular sialidase domain of the protein product is cleaved by ADAM10 and ADAM17 transmembrane proteases, and released into the bloodstream where it can function as an endocrine factor [256,261,262,264]. ADAM10/ADAM17-dependent cleavage of membrane-bound Klotho is under the control of insulin signaling, which when taken in consideration that soluble Klotho represses insulin signaling, points to the existence of a feedback loop to prevent unremitting insulin signaling [255,264]. While the membrane-bound form of Klotho serves as a coreceptor for fibroblast growth factor-23 (FGF23) and regulates phosphate and vitamin D metabolism, the secreted form neither binds to, nor serves as a coreceptor for FGF23 [256,265]. Rather, soluble Klotho serves as an enzyme capable of removing sugars and sialic acid residues from various membrane glycoproteins [253,256,261,262,263]. It is via the influence on membrane glycoproteins that soluble Klotho prolongs longevity.

### Regulation of Ion Channel Activity by Klotho

By removing glycan moieties from the extracellular side of the plasma membrane, soluble Klotho promotes the surface retention and activities of TRPV5 and TRPV6 ion channels [263,266,267,268,269]. Removal of terminal sialic acid residues allows the remaining sugars to bind lectins leading to channel crosslinking on the cell surface and diminished internalization [263]. Given that TRPV5 participates in systemic Ca^2+^ and phosphate homeostasis by functioning in the kidney [270], it is not clear whether Klotho influences longevity via these channels. Nevertheless, it is possible that hyperphosphatemia and hypercalcemia resulting from diminished TRPV5/TRPV6 function in the absence of Klotho could contribute to age-dependent vascular calcification and osteopenia [271]. A putative link to longevity can be gleaned from the demonstration that Klotho promotes the surface retention and overall activity of the Na^+^/K^+^ ATPase [272]. Given the relationship between neuronal excitability and longevity, and demonstrated roles for the Na^+^/K^+^ ATPase in the regulation of these parameters [10,11,12,16], soluble Klotho could influence lifespan by stabilizing this ATPase at the cell surface.

Alternatively, Klotho could influence animal longevity by its influence on cardiac function. Klotho counteracts cardiac arrhythmia by ensuring the cell surface expression of KCNQ1/KCNE1 K^+^ channels that are involved in cardiac repolarization [273]. Loss of soluble Klotho promotes cardiac arrhythmia owing to decreased activity of the KCNQ1/KCNE1 channels in cardiomyocytes. Cardioprotective roles of Klotho also stem from downregulation of TRPC6 channel activity [274,275]. TRPC6 channels have vital roles in stress-induced pathological cardiac hypertrophy and remodeling [274]. By attenuating TRPC6 conductance, Klotho ameliorates cardiotoxicity [274,276].

## 7. Roles for Ion Channels in Regulation of Longevity by Temperature

All physiological systems are under the control of temperature. While an increase in temperature accelerates the rates of biological reactions, a decrease in temperature has the opposite effect [277]. The inverse relationship between rates of metabolism and longevity is why lower body temperatures are associated with longer lifespans, whereas higher body temperatures are more common in short-lived individuals [278,279,280]. In homeotherms, interventions that influence rates of aging and lifespan, for instance caloric restriction, also tend to alter body temperature in a manner that is consistent between the aforementioned relationship between temperature and longevity [278,281]. Cold temperatures in homeotherms lead to the generation of heat in brown adipose tissue via UCP1 [101,102,103,104,105]. Given the involvement of UCP proteins in regulation of longevity (as discussed above), cold-induced uncoupling could constitute another mechanism by which low temperatures promote longevity. In agreement, overexpression of *Ucp2* in the hypocretin neurons of mouse hypothalamus extended the animals’ longevity by lowering their core body temperature [125].

In poikilotherms such as *C. elegans* and *Drosophila*, a decrease in ambient temperature promotes stress resistance and counteracts inflammatory signaling [282]. While it is tempting to speculate that the relationship between temperature and age-related pathology stems from the propensity of temperature to increase thermodynamic entropy [278,283], it is likely that additional levels of complexity bear upon this relationship. Indeed, flies cycled between hot and cold ambient temperatures live for as long as those that are reared at steady low ambient temperature [278,279,284]. These intriguing data suggested that the process of sensing a drop in ambient temperature is sufficient to impart the benefits of low temperature [278].

### 7.1. Thermosensitive Channels and Lifespan

**Figure 7:**
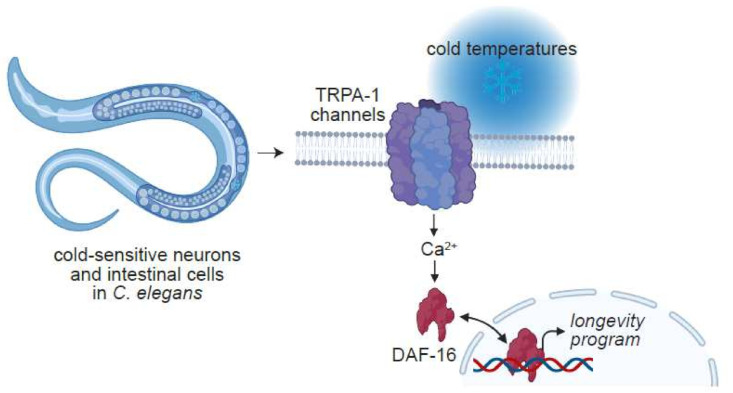
Effect of low-temperature on the lifespan of *C. elegans* is mediated by the cold-sensitive ion channel, TRPA-1. TRPA-1 is a cold sensitive ion channel that resides in the plasma membrane of *C. elegans* neurons and intestinal cells. Activation of the channels at 15 °C, results in cytosolic Ca^2+^ elevation and the activation of a signaling cascade that culminates in DAF-16/FOXO-mediated gene transcription, which in turn, promotes worm longevity. Image created with BioRender.com (accessed 27 January 2022).

In agreement with the notion that the act of sensing ambient temperature influences longevity, recent studies have shown that thermosensitive ion channels actuate the effects of temperature on lifespan. A pioneering study in *C. elegans* challenged the passive thermodynamic model of aging by demonstrating that a cold-sensitive TRP channel (TRPA-1) detects a drop in environmental temperature, and signals to the well-established modulator of longevity, DAF-16/FOXO [5,285,286] (Figure 7). *trpa-1* deficient adult worms exhibited significantly shorter lifespans when reared at a cool 15 °C, whereas no such difference between wild-type and *trpa-1* mutants was observed in animals raised at 25 °C [285,286]. These findings suggest that cold-induced extension of lifespan involves an ion channel that is directly activated by lower temperature rather than a general reduction in rates of metabolism (Figure 7).

Mice lacking the thermosensitive TRP channel, TRPV1, exhibit youthful metabolism and are relatively long-lived [287]. The regulation of lifespan via TRPV1 involves nuclear localization of the CREB-regulated transcriptional coactivator, CRTC1. In animals lacking TRPV1, CRTC1 is excluded from the nucleus, which results in diminished production of the neuropeptide CGRP and attendant improvement of metabolic health [287]. The relationship between CGRP and systemic metabolism is ensured by the repression of insulin release by CGRP [287]. In *C. elegans*, deletion of the TRPV channel genes, *ocr-2* and *osm-9*, led to the extension of lifespan [287,288]. Whether the activities of TRPV1, OSM-9, and OCR-2 in these contexts involve temperature remain to be addressed.

### 7.2. Heat-shock Response and Lifespan

While low temperature is associated with the extension of lifespan in many species, transient heat shocks in poikilotherms induce the expression of molecular chaperones that enforce proteostasis and promote longevity [289,290,291]. Interestingly, longevity-promoting effects of the heat-shock response in *C. elegans* are not cell autonomous, but rather stem from thermosensitive neurons that express cyclic nucleotide-gated (CNG) TAX-2/TAX-4 channels [292]. Deletion of guanylyl cyclase responsible for the activation of these CNG channels in thermosensitive neurons prevents the induction of a heat-shock response in other tissues [292]. The underlying mechanism involves the secretion of FMRFamide neuropeptide from the thermosensitive neurons, which greatly influences peripheral insulin signaling [293]. Furthermore, induction of the *C. elegans* heat shock factor, HSF-1, and the attendant effects on proteostasis can occur independently of a bona fide heat shock stimulus. Cholinergic neurotransmission at the neuromuscular junction leads to a Ca^2+^-dependent induction of the *hsf-1* in postsynaptic muscle cells via a process requiring the VGCCs and ryanodine receptors in the ER [294].

## 8. Regulation of Longevity by Channels Involved in Other Sensory Modalities

Besides temperature, many sensory modalities can modulate lifespan. In *Drosophila*, critical insights were obtained from the demonstration that deletion of the gene encoding a water-sensitive channel belonging to the amiloride-sensitive ENaC family of Na^+^ channels, *pickpocket 28* (*ppk28*), altered the metabolic status of animals, and thereby, extended lifespan [295,296,297]. Loss of *ppk28* improved systemic metabolism via the augmentation of neuronal signaling involving glucagon-related, adipokinetic hormone (AKH) [295]. Remarkably, the apparent lack of water—mimicked by the deletion of *ppk28*—triggers AKH-dependent alterations in metabolic pathways such as b-oxidation that produce molecular water, and in the process, extends lifespan [295].

In *C. elegans*, activity of sensory neurons, which is partially dependent on the ENaC channel MEC-4, is necessary for the protection of mitochondria from fragmentation in aged animals [298]. Furthermore, the TRPV channel, OSM-9, functions in sensory neurons to mediate the avoidance of hypertonic stress [299,300]. Deletion of *osm-9* led to enhanced proteostasis and survival of the worms when placed in hypertonic stress [300]. Deletion of another TRPV channel gene in *C. elegans*, *ocr-2*, also extended adult lifespan [287,288]. In *Drosophila*, loss of a similar TRPV channel, Inactive (Iav), which has been proposed to play roles in mechanosensation, is associated with reduced lifespan [301,302].

Relationships between lifespan and reproduction and/or food availability have been observed across taxa. In flies, detection of female pheromones via gustatory sensory neurons leads to reduction in stored nutrient reserves, greater sensitivity to starvation, and shorter lifespan in males [303]. This deleterious effect of female pheromone sensation on male longevity was observed only if the males were not allowed to mate, and mating reversed the effects of pheromone sensation on longevity. These findings indicate that the relationship sensory and reward circuits in the brain modulates aging and lifespan [303]. Although the involvement of ion channels in this axis of longevity have not been described, indirect evidence suggests the involvement of ENaC channels. Deletion of *ppk23* and *ppk25* result in diminished intensities of male courtship, likely due to altered responses to female pheromones [304,305]. It would be worth evaluating whether the absences of *ppk23* and *ppk25* influence the lifespan of the animals in a pheromone- and courtship-dependent manner. As was the case with female pheromones regulating male lifespan in fruit flies, food-derived odors shorten fly lifespan, and even deter the longevity-promoting effects of dietary restriction [306]. Therefore, deletion of the gene encoding, Or83b, an ionotropic odorant receptor that functions as a non-selective cation channel needed for the detection of food-derived odors, augmented stress resistance and extended animal lifespan [306,307,308].

## 9. Closing Remarks and Future Directions

Despite the plethora of ion channels and transporters that influence aging and longevity, holistic evaluation of the studies described here also reveal commonalities. One general principle is that mechanisms of ion transport constitute major regulatory axes in pathways that determine the rates of cellular or organismal aging. Some channels even function at the intersection of one or more hallmarks of aging. A better understanding of how these channels and transporters impact age-related loss of biological integrity could aid in the development of effective anti-aging strategies. Another theme that emerges from these studies is that homeostatic regulation of ion channel and transporter activity is the key to human healthspan. Any deviation of ion transport from a healthy optimum leads to elevated rates of mortality. Consequently, the trajectory of age-dependent loss of biological function likely varies greatly from one individual to the next. What we need, therefore, is the development of strategies that would enable personalized approaches to combat aging. A suite of drugs that restores ionic homeostasis in various tissues by appropriately modifying channel and transporter activities could be the key to translate biological insights into actionable therapeutic strategies. Future studies into the regulation of aging and longevity by ion channels and transporters will undoubtedly bring us closer to realizing this vision.

## Data Availability

Not applicable.
